# Mapping mHealth (mobile health) and mobile penetrations in sub-Saharan Africa for strategic regional collaboration in mHealth scale-up: an application of exploratory spatial data analysis

**DOI:** 10.1186/s12992-017-0286-9

**Published:** 2017-08-22

**Authors:** Seohyun Lee, Yoon-min Cho, Sun-Young Kim

**Affiliations:** 1 Department of Management, Policy and Community Health, University of Texas Health Science Center at Houston, School of Public Health, 1200 Pressler Street, Houston, TX 77030 USA; 2 Department of Public Health, Seoul National University, Graduate School of Public Health, 1 Gwanak-ro, Gwanak-gu, Seoul, 08826 South Korea; 3 Center for Global Health Research, Seoul National University, Graduate School of Public Health, 1 Gwanak-ro, Gwanak-gu, Seoul, 08826 South Korea

**Keywords:** mHealth, Exploratory spatial data analysis, Spatial autocorrelation, Sub-Saharan Africa

## Abstract

**Background:**

Mobile health (mHealth), a term used for healthcare delivery via mobile devices, has gained attention as an innovative technology for better access to healthcare and support for performance of health workers in the global health context. Despite large expansion of mHealth across sub-Saharan Africa, regional collaboration for scale-up has not made progress since last decade.

**Methods:**

As a groundwork for strategic planning for regional collaboration, the study attempted to identify spatial patterns of mHealth implementation in sub-Saharan Africa using an exploratory spatial data analysis. In order to obtain comprehensive data on the total number of mHelath programs implemented between 2006 and 2016 in each of the 48 sub-Saharan Africa countries, we performed a systematic data collection from various sources, including: the WHO eHealth Database, the World Bank Projects & Operations Database, and the USAID mHealth Database. Additional spatial analysis was performed for mobile cellular subscriptions per 100 people to suggest strategic regional collaboration for improving mobile penetration rates along with the mHealth initiative. Global Moran’s I and Local Indicator of Spatial Association (LISA) were calculated for mHealth programs and mobile subscriptions per 100 population to investigate spatial autocorrelation, which indicates the presence of local clustering and spatial disparities.

**Results:**

From our systematic data collection, the total number of mHealth programs implemented in sub-Saharan Africa between 2006 and 2016 was 487 (same programs implemented in multiple countries were counted separately). Of these, the eastern region with 17 countries and the western region with 16 countries had 287 and 145 mHealth programs, respectively. Despite low levels of global autocorrelation, LISA enabled us to detect meaningful local clusters. Overall, the eastern part of sub-Saharan Africa shows high-high association for mHealth programs. As for mobile subscription rates per 100 population, the northern area shows extensive low-low association.

**Conclusions:**

This study aimed to shed some light on the potential for strategic regional collaboration for scale-up of mHealth and mobile penetration. Firstly, countries in the eastern area with much experience can take the lead role in pursuing regional collaboration for mHealth programs in sub-Saharan Africa. Secondly, collective effort in improving mobile penetration rates for the northern area is recommended.

**Electronic supplementary material:**

The online version of this article (doi:10.1186/s12992-017-0286-9) contains supplementary material, which is available to authorized users.

## Background

During the past decade, the advancement of information and communication technology (ICT) has led to the emergence of mobile health (mHealth). In the global health context, mHealth can be defined as a healthcare delivery system that is carried out via mobile devices for better access to healthcare and support for performance of health workers [1]. Since its early development stage, the potential for an mHealth approach has been widely explored to empower public health functions for low- and middle-income countries (LMICs). In fact, the majority of mHealth programs are implemented in sub-Saharan Africa, where population health status is relatively poor, thus requiring more attention from a global health perspective [2, 3]. Despite the significant expansion of mHealth programs and access to mobile phones across sub-Saharan Africa over the past decade (2006–2016), cross-border regional collaboration for scale-up effort has rarely been initiated.

Previous studies on mHealth have addressed lack of scale-up effort, leading to limited evidence on cost-effectiveness, efficacy and feasibility [4]. A piecemeal approach to mHealth with small geographic coverage in sub-Saharan Africa can be myopic and dismiss the potential for scalability for the following two reasons. Firstly, small mHealth projects with low population coverage are not taking advantage of the economies of scale in mHealth implementation. Partnerships among different countries can be an enabler for cost reduction, specifically for fixed cost such as costs for technology, administration, personnel and promotional costs [5]. It can also be helpful in minimizing overspending and the costs incurred by a system’s incompatibility and reengineering [6]. Secondly, cross-border mHealth programs can play a key role in surveillance, monitoring and control of communicable and non-communicable diseases through mobile data collection and education. Collecting real-time information through mHealth is critical to disease preparedness and response as well as understanding the dynamics of the epidemiology within sub-Saharan Africa. The recent Ebola outbreak in West Africa is a case in point [7]. Sierra Leone, in collaboration with the technology company IBM and the nation’s largest mobile network carrier AirTel, initiated a disease-mapping system in 2014 by asking local people to report Ebola to the government via free text messages [8]. Additionally, Sierra Leone worked with the Red Cross and AirTel to send educational text messages about hygiene measures in the areas most susceptible to the pandemic. However, as is the case for Sierra Leone, most of these mHealth programs are implemented at the national level.

The importance of collaboration among neighboring countries has been well understood by researchers and many stakeholders in Africa. In their quantitative research, Tyler and Gopal identified geographic clusters in the levels of development in sub-Saharan Africa and how clustering can contribute to formulating policy in sub-Saharan Africa [9]. Particularly, they identified the following: western and central African clusters had low levels of development, the eastern coast of central sub-Saharan Africa had higher income and a well-educated population, and landlocked countries had lower life expectancy, and so on [9]. Their findings have implications in the regional collaboration approach for mHealth according to different stages of development and experience based on geography. Historically, Africa has striven for regional integration for economic cooperation by geography as exemplified by the Economic Community of West African States (ECOWAS), the Common Market for Eastern and Southern Africa (COMESA) and the Economic Community of Central African States (ECCAS) [10]. In terms of regional cooperation for public health, WHO Regional Office for Africa’s Inter-country Support Teams for Central Africa (10 countries), Eastern and Southern Africa (20 countries) and West Africa (17 countries) work collaboratively to facilitate “technical support to countries for scaling up proven public health interventions” [11].

The first research question of the study was whether the total number of mHealth programs in a country are correlated with those of its neighboring countries. Using the two indicators of spatial autocorrelation −Global Moran’s I that measures the degree of spatial correlation of a variable with itself across the study region and Local Indicator of Spatial Association (LISA) that measures Moran’s I at the local level−, the aim of the study was to investigate spatial heterogeneity and local clustering of the number of mHealth programs implemented between 2006 and 2016 among sub-Saharan African countries [12]. The key idea is based on Tobler’s first law of geography that states “everything is related to everything else, but near things are more related than distant things” [13, 14]. If there is a spatial autocorrelation in the total number of mHealth projects implemented in each country, then the null hypothesis of spatial randomness is rejected. This in turn leads to the violation of a statistical assumption that values of observations in each geographic unit are independent.

In addition to spatial analysis on the mHealth implementation effort, the second research question to be answered in the study was whether the level of mobile phone penetration per 100 population in a country is correlated with that of its neighboring countries. Since the level of mobile penetration in each country is a key technical component for the expansion of mHealth in the region, it is meaningful to examine any spatial disparities in mobile subscription rates as well as the number of mHealth programs implemented in each country.

Considering the importance of collaboration for mHealth in global health, recent mHealth literature has addressed some spatial patterns. Deglise et al. published a systematic review on short messaging service (SMS) interventions for disease prevention and demonstrated that these mHealth projects were concentrated mostly in South Africa, Kenya and India [15]. Also, Gorski et al. (2016) found from their meta-analysis that globally, most mHealth projects are located in sub-Saharan Africa [16]. According to Njoroge et al. (2017), the growth of mHealth in sub-Saharan Africa is particularly outstanding in Kenya [17]. However, former studies have not attempted to quantitatively and systematically assess the mHealth and mobile penetrations in sub-Saharan Africa region. Therefore, this study systematically collected information on mHealth programs and examined their spatial distribution. In doing so, the study aimed to identify spatial gaps and disparities in both mHealth implementation effort and mobile subscription rates for the past decade. Potentially, the study can provide guidance on strategic regional collaboration for mHealth implementation by identifying inequity and resource reallocation opportunities for stakeholders including governments, donors, industry, international organizations, NGOs (non-governmental organizations) and other decision making entities.

## Methods

### Study setting

Sub-Saharan Africa is the region south of the Sahara desert and is located on the African continent. Known for its heavy burden of communicable, maternal, neonatal and nutritional diseases, sub-Saharan Africa has been suffering from limited access to care and poor health status compared to other continents [18]. In terms of mHealth interventions, sub-Saharan Africa has been considered as a region where applicability and potential of mHealth is promising with growing wireless network coverage and high mobile phone subscription rates [3, 19]. In 2015, the region’s average mobile cellular subscription rate was 82.9 per 100 people [20].

Table 1 illustrates socioeconomic and geographic information for all 48 sub-Saharan African countries. According to the World Bank and the United Nations Statistics Division, there are 48 countries located in the sub-Saharan African region, including 17 countries in the eastern Africa (Burundi, Comoros, Eritrea, Ethiopia, Kenya, Madagascar, Mozambique, Mauritius, Malawi, Rwanda, Somalia, South Sudan, Seychelles, Tanzania, Uganda, Zambia, Zimbabwe), 16 in western Africa (Benin, Burkina Faso, Cote d’Ivoire, Cabo Verde, Ghana, Guinea, the Gambia, Guinea-Bissau, Liberia, Mali, Mauritania, Niger, Nigeria, Senegal, Sierra Leone and Togo), 9 in middle Africa (Angola, Central African Republic, Cameroon, Democratic Republic of the Congo, Republic of the Congo, Gabon, Equatorial Guinea, Sao Tomoe and Principe and Chad), 5 in southern Africa (Botswana, Lesotho, Namibia, Swaziland and South Africa) and 1 in northern Africa (Sudan) [21, 22]. Among these, 6 countries (Comoros, Cabo Verde, Madagascar, Mauritius, Sao Tome and Principe and Seychelles) are island nations adjacent to the Atlantic and Indian Ocean.

**Table 1:** Country characteristics for all 48 sub-Saharan Africa countries by sub-region

Geographic Region	No.	Country	Population (2015)	GNI per capita, PPP (current US$, 2015)	Income group^a^	Adult literacy rate (15+ years, both sexes, %, 2015)	Healthy Life Expectancy at Birth (years, 2015)
Eastern Africa	1	Burundi	10,199,270	280	L	85.5	52.2
	2	Comoros (insular)	777,424	790	L	78.1	55.9
	3	Eritrea	4,474,690^b^	520^b^	L	73.8	55.7
	4	Ethiopia	99,873,033	600	L	49.0	56.1
	5	Kenya	47,236,259	1310	LM	78.0	55.6
	6	Madagascar (insular)	24,234,088	420	L	64.7	56.9
	7	Malawi	17,573,607	340	L	66.0	51.2
	8	Mauritius (insular)	1,262,605	9780	UM	90.6	66.8
	9	Mozambique	28,010,691	590	L	58.8	49.6
	10	Rwanda	11,629,553	710	L	71.2	56.6
	11	Seychelles (insular)	93,419	14,680	H	95.3	65.5
	12	Somalia	13,908,129	NA	L	NA	47.8
	13	South Sudan	11,882,136	820	L	32.0	49.9
	14	Tanzania	53,879,957	910	L	80.4	54.2
	15	Uganda	40,144,870	680	L	73.8	54
	16	Zambia	16,100,587	1560	LM	85.1	53.7
	17	Zimbabwe	15,777,451	960	L	86.9	52.1
	Average		24,536,442	2295.3	-	73.1	54.9
Western Africa	18	Benin	10,575,952	870	L	38.4	52.5
	19	Burkina Faso	18,110,624	650	L	37.7	52.6
	20	Cabo Verde (insular)	532,913	3150	LM	88.5	64.2
	21	Cote d’Ivoire	23,108,472	1490	LM	43.3	47
	22	Gambia, The	1,977,590	450	L	55.6	53.8
	23	Ghana	27,582,821	1470	LM	76.6	55.3
	24	Guinea	12,091,533	490	L	30.5	51.7
	25	Guinea-Bissau	1,770,526	610	L	59.8	51.5
	26	Liberia	4,499,621	380	L	47.6	52.7
	27	Mali	17,467,905	760	L	33.1	51.1
	28	Mauritania	4,182,341	1230	LM	52.1	55.1
	29	Niger	19,896,965	390	L	19.1	54.2
	30	Nigeria	181,181,744	2870	LM	59.6	47.7
	31	Senegal	14,976,994	980	L	55.6	58.3
	32	Sierra Leone	7,237,025	550	L	48.4	44.4
	33	Togo	7,416,802	540	L	66.5	52.8
	Average		22,038,114	1055	-	50.8	52.8
Middle Africa	34	Angola	27,859,305	4070	UM	71.2	45.9
	35	Cameroon	22,834,522	1350	LM	75.0	50.3
	36	Central African Republic	4,546,100	360	L	36.8	45.9
	37	Chad	14,009,413	880	L	40.0	46.1
	38	Congo, Dem. Rep.	76,196,619	430	L	77.2	51.8
	39	Congo, Rep.	4,995,648	2350	LM	79.3	56.6
	40	Equatorial Guinea	1,175,389	9190	UM	95.2	51.3
	41	Gabon	1,930,175	8010	UM	83.2	57.2
	42	Sao Tome and Principe (insular)	195,553	1700	LM	91.7	59
	Average		17,082,525	3148.9	-	72.2	51.6
Southern Africa	43	Botswana	2,209,197	6640	UM	88.2	56.9
	44	Lesotho	2,174,645	1300	LM	79.4	46.6
	45	Namibia	2,425,561	5260	UM	90.8	57.5
	46	South Africa	55,011,977	6090	UM	94.6	54.4
	47	Swaziland	1,319,011	3130	LM	87.5	50.9
	Average		12,628,078	4484	-	88.1	53.3
Northern Africa	48	Sudan	38,647,803	2000	LM	58.6	55.9

^a^Income group- *H* high income county, *UM* upper-middle income country, *LM* lower-middle income country, *L* low income country

^b^data from 2011

Currently, the World Bank defines a low income country whose GNI (gross national income) per capita was $1025 or less in 2015; 90% of the world’s low income countries are located in sub-Saharan Africa [22]. In fact, there is only 1 high income country in this region (Seychelles). The eastern region has 1 high income country, 1 upper-middle income country, 2 lower-middle income countries and 13 low income countries. The western region has 5 lower-middle income countries and 11 low income countries. In the middle region, there are 3 upper-middle income countries, 3 lower-middle income countries and 3 low income countries. In southern region, 3 countries belong to upper-middle income group and 2 are lower-middle income countries. Sudan, in the north, is a lower-middle income country. The average GNI per capita for each region (current US$) in 2015 was: Eastern region− 2295.3, western region− 1055, middle region − 3148.9, South − 4484 and North − 2000.

The average population size is not too different between the east and the west 24,536,442 and 22,038,114, respectively; however, population gaps exist between the eastern Africa countries and the western Africa countries. For example, the average healthy life expectancy at birth in 2015 was 52.8 years (STD 4.6, Min 44.4, Max 64.2) for the western Africa countries whereas it was 54.9 years (STD 5, Min 47.8, Max 66.8) among the eastern Africa countries [23]. Also, the average adult literacy rate in 2015 was 50.8% (STD 17.6, Min 19.1, Max 88.5) in the west while it was 73.1% (STD 16.3, Min 32, Max 95.3) in the east [23, 24].

### Data sources

For the systematic data collection on the number of mHealth programs implemented in each sub-Saharan Africa country between 2006 and 2016, the following data sources were used: The WHO eHealth database, the United States Agency for International Development (USAID) mHealth database and the mHealth Working Group Inventory of Projects (Johns Hopkins University) were accessed. The variety of databases provides a comprehensive list of mHealth programs with project descriptions and basic information. To increase sensitivity, non-mHealth or non-eHealth specific databases were also accessed including the World Bank Projects & Operations database, the African Development Bank (AfDB) Projects & Operations database, the UK Department for International Development (DFID) Development Tracker, the Canadian International Development Agency (CIDA) and the Center for Health Market Innovation (CHMI) database. Since these databases contain mHealth projects as well as other international development projects, several search terms were used to secure specificity. Examples of search terms include mHealth, mobile health, mobile phone, cell phone, cellular phone, smart phone, mobile device, wearable device, tablet, PDA (personal digital assistant), SMS, Multimedia Message Service (MMS), text message, phone call and email.

From the above mentioned data sources, a comprehensive dataset was created after selecting mHealth projects based on inclusion/exclusion criteria. To be included in the study, mHealth projects must have been implemented between 2006 and 2016 in one of the sub-Saharan Africa countries. Other eHealth projects that do not explicitly involve the use of mobile devices were excluded. Geographic Information System (GIS) data was obtained from the GADM database (Global Administrative Areas), which is an open-source repository of spatial data developed by researchers at the University of California at Davis, the University of California at Berkeley and others [25]. Historical data on the mobile cellular subscriptions per 100 population and other country characteristics were obtained from publicly available data on the World Bank and WHO websites [23, 24].

### Exploratory spatial data analysis

This study attempted to investigate if the number of mHealth programs implemented in a country is spatially correlated with that in its neighboring countries, using exploratory spatial data analysis (ESDA). The same process has been performed for the average mobile cellular subscriptions per 100 population between 2006 and 2015. The average mobile subscription rate during the past decade was used for the analysis because it represents not only the current situation but the overall mobile network coverage trend for each country. To ensure that the average mobile subscriptions per 100 population between 2006 and 2015 were not statistically different from the current situation for all 48 sub-Saharan Africa countries, a *Friedman* test was performed between the rank order measures of the average mobile subscription rate per 100 population in 2006 and that in the year 2015. The *Friedman* chi-square value was 0.19 with a *p*-value of 0.67, thus the two ranks were not statistically different.

In conducting an exploratory spatial data analysis, it is possible to identify a local clustering within the study region. For the purposes of our study, two measures of spatial autocorrelation were investigated: Moran’s I and Local Indicator of Spatial Association (LISA). Moran’s I is used to measure global spatial autocorrelation by looking at the correlation between the variable of interest *y* (in this case, the total number of mHealth programs or mobile cellular subscriptions per 100 people) and the spatial lag of that variable *y* [26, 27]. Spatial lag of a variable *y* is derived from the average value of *y* for all the neighboring locations. The slope of the least squares linear regression line between *y* and lag-*y* is referred to as Moran’s I. The decision on whether a location *j* is a neighbor of a location *i* or not is introduced in the data by a weights matrix *w*_*ij*_ (*i* ≠ *j*) [12]. To decide what constitutes as a neighbor, a weights matrix had to be defined. There are several ways to designate spatial weights matrix based on contiguity or distance. This study adopted a weights matrix using queen contiguity where countries were considered neighbors when they shared a common boundary or a point [28]. *w*_*ij*_ was equal to 1 if country *i* and country *j* were neighbors and 0 if they were not neighbors according to the queen’s contiguity rule. Choosing a weights matrix scheme is often arbitrary and determined by the research topic and outcome of interest. This weights matrix, queen contiguity, was chosen for this study based on the literature review on exploratory spatial data analysis for spatial trends of incidence [26, 29–31]. The Moran’s I generally ranges from −1 to 1, with 1 having a positive spatial autocorrelation and −1 having a negative spatial autocorrelation. If Moran’s I is 0 then there is no spatial autocorrelation, indicating spatial randomness.

LISA was evaluated to find out the spatial clustering for each location [29]. While Moran’s I is a global measure of spatial autocorrelation, LISA is a local Moran’s I and therefore, the sum of the LISA is proportional to a global indicator, Moran’s I [32]. However, Moran’s I has a major limitation in that it cannot identify meaningful clusters or spatial patterns at a local scale. On the contrary, LISA enabled us to identify hot spots where the variable of interest *y*_*i*_ (the total number of mHealth programs or mobile cellular subscriptions per 100 people) has higher value than average for the entire study region $\overline{y}$ and where the same held true for its neighbors [30]. This is known as a high-high association or a hot spot. Similarly, in the same manner, cold spots or low-low associations were able to be identified using LISA. The proximity of the locations *i* and *j* is also represented by weights matrix *w*_*ij*_ defined by the queen contiguity rule (*i* ≠ *j*) [12]. ArcGIS software V10.5 was used for statistical analysis and mapping.

## Results

### mHealth programs in sub-Saharan Africa

From our systematic data collection, the total unique number of mHealth programs implemented in sub-Saharan Africa between 2006 and 2016 was 487 (any duplicates were removed and any program that was implemented in multiple countries was counted separately for each country). Of these mHealth programs counted, the eastern and western regions had 287 and 145 for the past decade, respectively. More specifically, Kenya, Uganda and Tanzania in the eastern region were with the most mHealth programs implemented between 2006 and 2016 (71, 54, and 50, respectively).

**Fig. 1:**
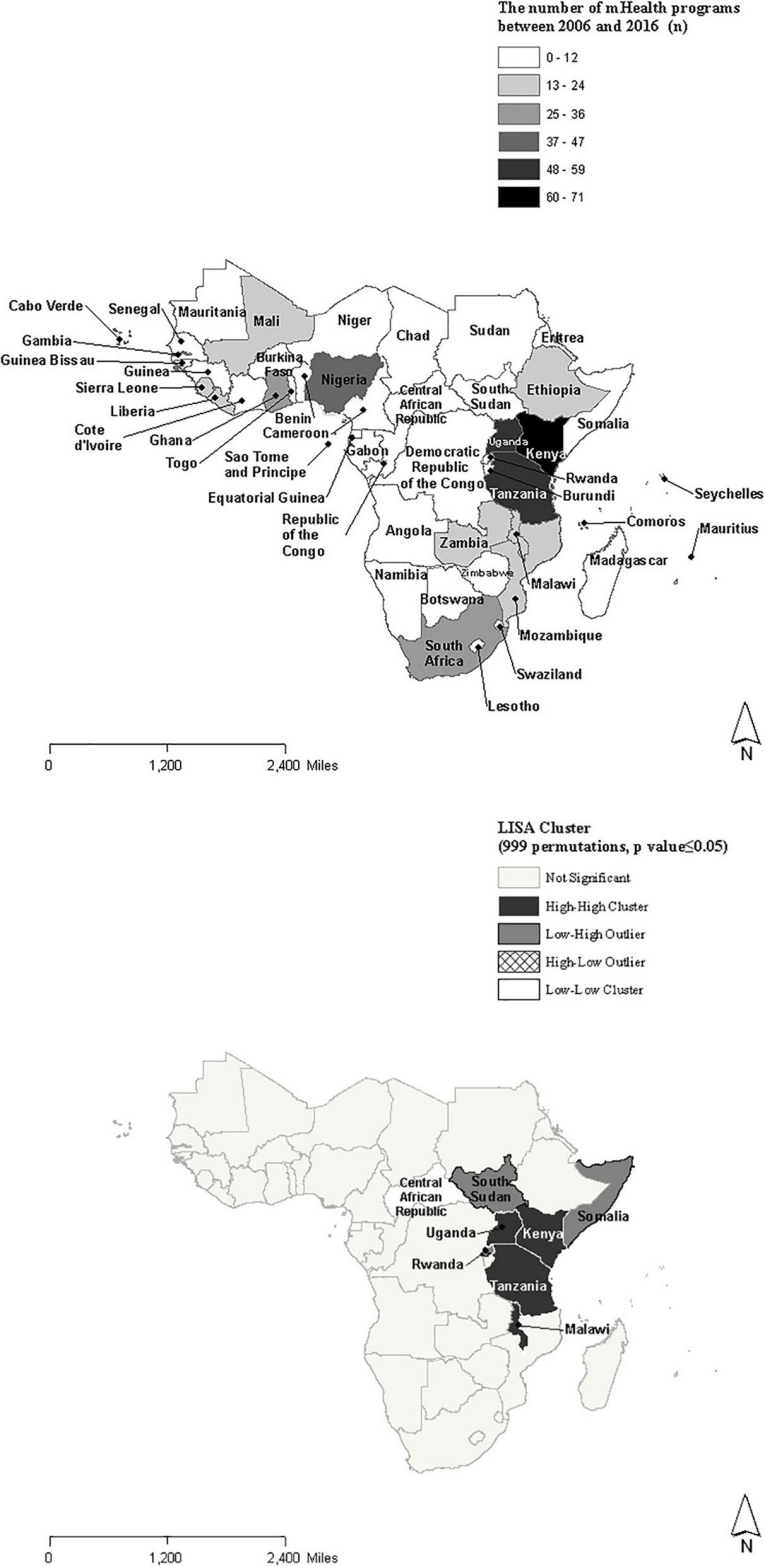
a and b Choropleth map of the number of mHealth programs and corresponding LISA clusters

Global Moran’s I for mHealth programs in sub-Saharan Africa was 0.16 with a pseudo *p*-value = 0.07, indicating an insignificant level of spatial autocorrelation at the global level. Figure 1a and b illustrate the choropleth map on the count of mHealth programs in each country and the corresponding statistically significant LISA clusters. For the LISA analysis, the significance level was filtered at a pseudo *p*-value = 0.05 and 999 permutations were performed for sensitivity analysis.

Despite relatively low levels of global Moran’s I, the analysis of LISA clusters allowed us to detect hot spots or high-high associations where a certain high value in a country correlated with a high value in its neighboring countries. Likewise, cold spots (low-low association), low-high associations and high-low associations were identified by LISA. As for mHealth programs, hot spots were identified along the eastern coastline, Kenya, Tanzania, Malawi and Uganda. South Sudan, Somalia and Rwanda are identified as low-high associations particularly because they had relatively few mHealth programs implemented but are contiguous to Uganda, Kenya or Tanzania where large numbers of mHealth programs had been identified. The Central African Republic was considered a low-low association due to limited experience with mHealth by both its neighboring countries and itself.

### Mobile subscriptions per 100 population in sub-Saharan Africa

**Fig. 2:**
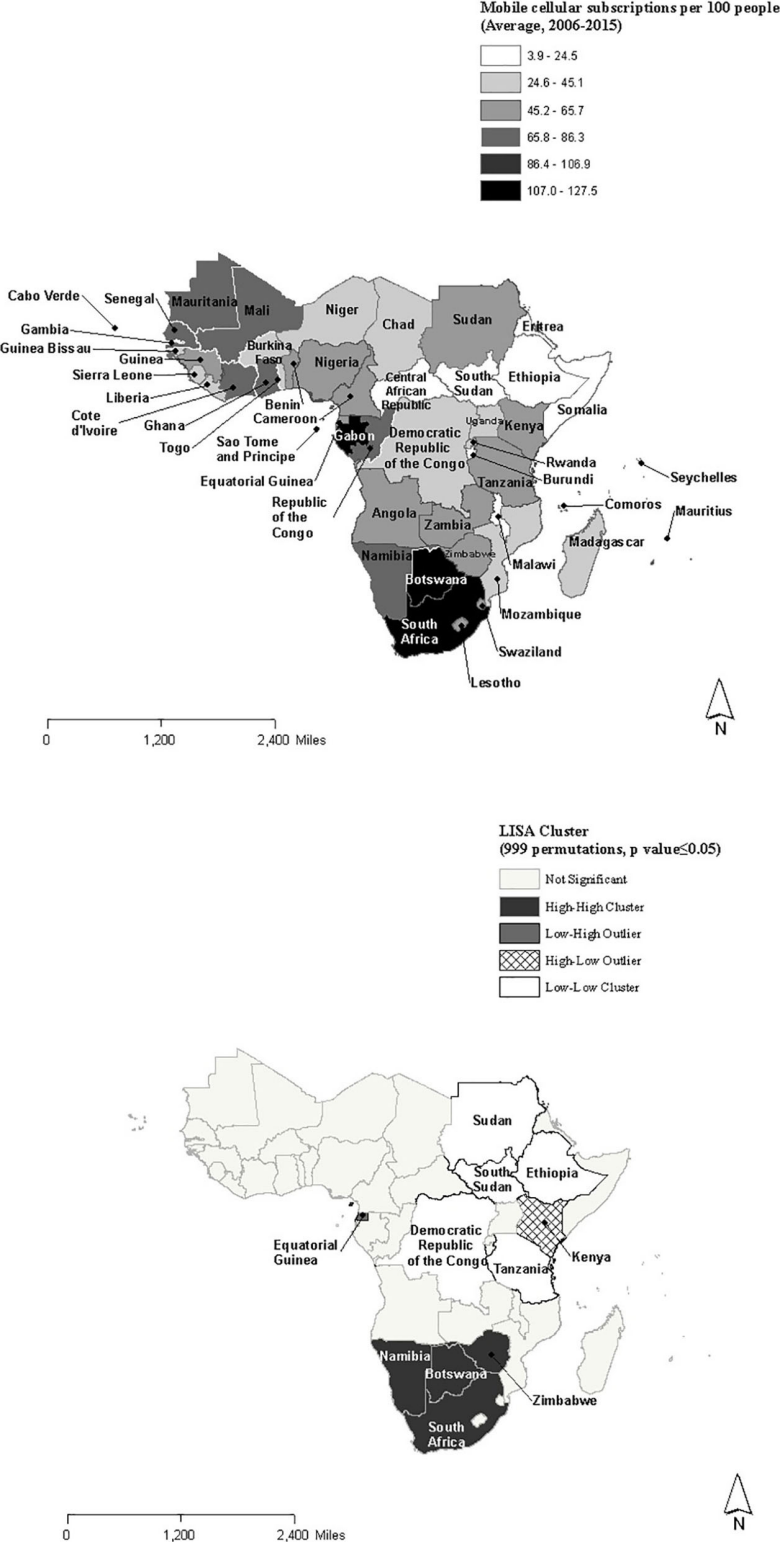
a and b Choropleth map of the mobile subscriptions per 100 people and corresponding LISA clusters

Global Moran’s I for average mobile subscription rates per 100 population between 2006 and 2015 is 0.29 with a pseudo *p*-value = 0.01, indicating the presence of geographic clustering to some extent. Figure 2a and b present the choropleth map of average mobile subscriptions per 100 population between 2006 and 2015 and the corresponding LISA clusters. The same significance filter and sensitivity analysis were applied as the LISA cluster analysis for mHealth programs. Average mobile penetration rates per 100 population were greater than 100 in Seychelles, Gabon, Botswana, South Africa and Mauritius for the past decade. In contrast, some of the countries with the lowest average mobile subscription rates had less than 50 subscriptions per 100 people during 2006 and 2016.

LISA analysis for average mobile subscriptions per 100 population between 2006 and 2015 identified extensive cold spots across northeastern and central parts of the sub-Saharan Africa (Sudan, South Sudan, Ethiopia, Democratic Republic of the Congo and Tanzania). In addition, 1 high-low association (Kenya) and 1 low-high association (Equatorial Guinea) were identified for average mobile subscription rates per 100 population. In the southern area, Namibia, Botswana, Zimbabwe and South Africa were identified as having had high-high associations where mobile subscriptions were high for themselves as well as for their neighbors.

## Discussion

### Toward strategic regional collaboration for mHealth

Although it is well known that using mobile technology in health service delivery can be effective in resource-limited settings such as sub-Saharan Africa, challenges still remain in terms of scale-up efforts [4]. In addition, setting up reliable technology and infrastructure is essential to ensure sustainability of mHealth programs. Since sub-Saharan Africa is the region where investment in mHealth has been rapidly growing, it is the appropriate time to establish regional collaboration strategies to facilitate a synergy effect. To that end, this study attempted to lay a groundwork by investigating spatial distribution on the number of mHealth programs and mobile subscription rate. Specifically, the study identified geographic areas with relatively more/less mHealth programs or higher/lower mobile cellular penetration rates.

The main findings of our analysis suggest the following: Firstly, countries in the east of sub-Saharan Africa have a comparative advantage with more experiences in mHealth implementation than their counterparts, specifically for hot spot countries such as Kenya, Tanzania, Malawi and Uganda. These hot spots imply that they have the potential to take the lead role in moving towards a collaborative mHealth approach for scale-up in sub-Saharan Africa. Furthermore, low-high association countries such as South Sudan, Somalia and Rwanda can take advantage of their geographic location by closely collaborating with hot spot neighboring countries that can share knowledge and experience. For the low-low association case of Central African Republic, the inexperience in mHealth for itself and its neighboring countries coincides with overall underdevelopment in landlocked countries across the continent [9]. In fact, landlocked locations have long been blamed for their contribution to high transaction costs and less opportunities for economic growth [10].

Secondly, middle and northeastern areas of sub-Saharan Africa have been cold spots for mobile cellular penetration rates for the past decade. This phenomenon resonates with the disadvantages, mentioned earlier, attributed to the landlocked locations. Among the five countries identified as cold spots for mobile cellular subscription rates, South Sudan and Ethiopia are landlocked countries and the Democratic Republic of the Congo is also mostly landlocked. Our findings are in line with other research that pointed out the lower level of expansion in mobile coverage for the landlocked countries in the central and western region of Africa [33]. Kenya is identified as a high-low country that shows high mobile penetration rates but is surrounded by countries with low mobile penetration rates. Interestingly, Tanzania is one of the countries with the largest numbers of mHealth programs implemented between 2006 and 2016, but it is a cold spot for its average mobile subscription rate between 2006 and 2015. Tanzania makes a good case for further analysis on how it dealt with relatively low mobile subscription rates when implementing such a large number of mHealth programs. Hot spot countries in the south (Namibia, Botswana, Zimbabwe and South Africa) can lead the discussion on the regional collaboration strategies in improving mobile network coverage for effective mHealth scale-up.

### Limitations

Some of the limitations of this study include the following. Firstly, the number of mHealth programs is aggregated to the country level, making it difficult to identify a detailed regional distribution across the border. As for the mobile subscription rate per 100 population data, it may overestimate or underestimate the number of actual users of mobile phones because ownership of multiple subscriber identity module (SIM) cards by an individual is very common while sharing mobile phones among friends or family is also common in Africa [33]. Future spatial analysis on mHealth and mobile subscriptions can address this issue by looking at the data on a higher level of granularity. Secondly, there can be a risk of publication bias in that the mHealth project information data available online may not represent all mHealth implementation efforts within a country. To minimize this possibility, the study combined official data repositories from various organizations. Finally, this study can be further expanded for future research that takes into account for the temporal component of the data to investigate the trend in mHealth growth and the change in mobile technology penetration rates.

Despite the limitations, our study was the first to examine spatial heterogeneity in mHealth implementation effort along with the mobile subscription rates within the sub-Saharan Africa region. Identifying regional inequity in public health interventions such as mHealth can play a key role in informing stakeholders for strategic regional cooperation in terms of future resource allocation decision and new investment opportunities. In particular, the LISA analysis from our study demonstrates that the spatial differences are not randomly distributed but have some spatial pattern within the sub-Saharan Africa region. As the result of our study indicates, areas along the eastern coastline with much experience in mHealth can be a regional hub for collaboration whereas the northeastern and central parts of sub-Saharan Africa need more attention for improving access to mobile phones.

### Future directions

To go one step further, more research should be conducted on developing specific strategies for regional collaboration in mHealth and enhanced mobile penetration. In particular, there are several clusters of countries in sub-Saharan Africa that share common languages or common mobile network providers, which can be beneficial in regional collaboration for scaling up mHealth. Stakeholders from hot spot countries should actively participate in the discussion for plan of action. In addition, a root cause analysis should be performed on why cold spot countries lag behind in terms of mHealth implementation and access to mobile phones. In scaling up mHealth and tackling disparities in mobile penetration rates, regional collaboration or regional networks for mHealth and mobile technology can contribute to creating opportunities for exchanging hands-on knowledge and lessons learned among countries with different levels of experience.

## Conclusions

Overall, the exploratory spatial data analysis on the mHealth implementation effort and mobile subscription rates has significant implications on developing strategies for regional collaboration and identifying disparities within the sub-Saharan Africa region. The spatial distribution of mHealth programs and mobile penetration rates identified from the study can be useful in decision making for future scale-up efforts in mHealth and for better access to mobile phones in sub-Saharan Africa. According to the World Bank, there were more than 500 mHealth projects worldwide in 2011 alone and the number is still growing [34]. Therefore, strategies to minimize duplicate effort and scale up current initiatives must be implemented [35]. In this light, Howitt et al. suggested the establishment of one organization responsible for maintaining mHealth trials registry and assessments [36].With a growing number of mHealth initiatives along the eastern coastline of sub-Saharan Africa, those countries with more experience can take the lead role in a collective scale-up effort. Additionally, the northeastern and central part of sub-Saharan Africa should be given more attention in terms of access to mobile phones, which is an integral part of scaling up the mHealth initiative.

## Additional file

Additional file 1: A complete list of mHealth programs for all 48 sub-Saharan Africa countries between 2006 and 2016 from 8 data sources (DOCX 90 kb)

## Abbreviations

AfDB: African Development Bank
CHMI: Center for Health Market Innovation
CIDA: Canadian International Development Agency
COMESA: Common Market for Eastern and Southern Africa
DFID: UK Department for International Development
ECCAS: Economic Community of Central African States
ECOWAS: Economic Community of West African States
ESDA: Exploratory Spatial Data Analysis
GADM: Global Administrative Areas
GIS: Geographic Information System
GNI: Gross National Income
ICT: Information Communication Technology
LISA: Local Indicator of Spatial Association
LMICs: Low- and Middle-Income Countries
mHealth: Mobile Health
MMS: Multimedia Message Service
NGOs: Non-governmental Organizations
PDA: Personal Digital Assistant
SIM: Subscriber Identity Module
SMS: Short Message Service
USAID: United States Agency for International Development
WHO: World Health Organization
