# Supplementary material for: Mapping mHealth (mobile health) and mobile penetrations in sub-Saharan Africa for strategic regional collaboration in mHealth scale-up: an application of exploratory spatial data analysis
Source: Global Health. 2017 Aug 22;13:63. doi: 10.1186/s12992-017-0286-9 (PMC5568212; doi:10.1186/s12992-017-0286-9)
Supplement: Additional file 1: — A complete list of mHealth programs for all 48 sub-Saharan Africa countries between 2006 and 2016 from 8 data sources (DOCX 90 kb) [file 12992_2017_286_MOESM1_ESM.docx]

Additional file 1

A complete list of mHealth programs for all 48 sub-Saharan Africa countries between 2006 and 2016 from 8 data sources

| **No** | **Title** | **Description** | **Country** | **Source†** | **Start year** |
| --- | --- | --- | --- | --- | --- |
| 1 | Africa Indoor Residual Spraying (AIRS) | Abt developed a mobile app to improve environmental compliance by providing a phone-based checklist | Angola, Benin, Burkina Faso, Ethiopia, Ghana, Liberia, Madagascar, Mali, Mozambique, Nigeria, Rwanda, Senegal, Zimbabwe | Hopkins | 2013 |
| 2 | Strengthening Pharmaceutical Systems (SPS) | PMI (Presidnet's Malaria Initiative) data collection, aggregation and reporting | Angola, Benin, Burundi, Kenya, Namibia, Uganda | Hopkins | 2009 |
| 3 | Accelerating the Reduction of Morbidity of and Mortality of Malaria (ARM3)'s iCCM mHealth Initiative | An mHealth initiative designed to facilitate the reporting and exchange of information between Community Health Workers (CHWs) and health facilities | Benin | Hopkins | 2013 |
| 4 | CORE Group CommCare mHealth Collaborative | The use of mobile technology by Community Health Workers (CHW) consisting of developing a family planning module that includes the use of images, audio and video clips for routine family planning counseling | Benin | Hopkins | 2012 |
| 5 | VaxTrac | A computer/tablet-based vaccine management system that incorporates the needs of end users, including health workers and mothers, as well as providing a useful platform for the Benin Ministry of Health | Benin | WHO | 2012 |
| 6 | CommCare for Antenatal Care Services | An easily customizable mHealth platform for health workers that tracks and supports their interactions with patients | Benin, Ethiopia, Ghana, Kenya, Malawi, Mozambique, Nigeria, Senegal, Sierra Leone, South Africa, Sudan, Tanzania, Zambia | USAID | 2013 |
| 7 | UBSOM mLearning Initiative | mLearning Initiaitive at the University of Botswana involves mHealth with regard to the introduction of handheld tablet computers in medical education, bedside clinical practice and teaching | Botswana | Hopkins | 2012 |
| 8 | Africa Teledermatology Project | Long distance consultation to provide dermatology support to local physicians, dermatologists, and health care workers in hospitals and clinics using computers, medical devices and mobile phones | Botswana, Burkina Faso, Lesotho, Malawi, Swaziland, Uganda | WHO | 2007 |
| 9 | Peek Vision | The portable eye examination kit consists of a mobile app and clip-on hardware | Botswana, Kenya, Mali | WHO | 2013 |
| 10 | Daktari CD4 | Providing a CD4 cell counting system that is portable and robust enough to be used anywhere, from a doctor’s office to the most remote settings | Botswana, Kenya, South Africa, Uganda | CHMI | 2014 |
| 11 | Airtel Insurance with MicroEnsure | Sustainable health microinsurance at scale (Airtel rewards loyal customers who spent a minimum airtime with free insurance) | Burkina Faso, Ghana, Kenya, Madagascar, Malawi, Nigeria, Niger, Zambia, | USAID | 2014 |
| 12 | Human Network International (HNI) | Provide organizations and individuals the opportunity to use ICT for health purposes including HIV, Malaria, Family planning, Maternal and child health, etc. | Burkina Faso, Ghana, Madagascar, Malawi, Mozambique, Nigeria, Uganda, Zambia | Hopkins | 2011 |
| 13 | Burundi Public Health Laboratory Networking Project | Operational research and knowledge sharing activities including use of mobile phone technologies for surveillance reporting of endemic diseases | Burundi | World Bank | 2012 |
| 14 | ExTBCam | An open-source solution for tuberculosis laboratory data management where data will primarily be entered via laptops | Cameroon | Hopkins | 2012 |
| 15 | Population Services International (PSI) | A mobile phone based data transmission system | Cameroon | Hopkins | 2012 |
| 16 | Medic Mobile | Text messages enable real-time communication between community health workers (CHWs) and their supervisors | Cameroon, Democratic Republic of the Congo, Ethiopia, Kenya, Malawi, Mali, Nigeria, Senegal, Tanzania, Uganda | CHMI | 2009 |
| 17 | SMS for Life | Track weekly stock levels for essential malaria medicines at public health facilities by mobile phones, SMS messages, the Internet and electronic mapping technology | Cameroon, Democratic Republic of the Congo, Ghana, Kenya, Tanzania | WHO | 2010 |
| 18 | Hang-up and Track | Increasing bednet distribution accountability and usage in households through mobile data collection | Democratic Republic of the Congo | USAID | 2014 |
| 19 | President’s Malaria Initiative (PMI) Expansion Project in DRC | The President’s Malaria Initiative (PMI) Expansion Project (PMI-EP) integrated mobile technology including SMS, web forms, basic handsets and smartphones | Democratic Republic of the Congo | Hopkins | 2013 |
| 20 | FioNet | Mobile diagnostics for rapid diagnostic tests (RDTs) integrated with cloud information services | Democratic Republic of the Congo, Ghana, Tanzania, Sierra Leone | USAID | 2012 |
| 21 | Healthy Entrepreneurs | Applications on the tablet are used for providing health awareness, stock management and data collection | Democratic Republic of the Congo, Uganda | CHMI | 2011 |
| 22 | Basic Emergency Obstetric and Newborn Care (BEmONC ) | Using SMS to support health worker learning/performance | Ethiopia | Hopkins | 2012 |
| 23 | Enat Messenger for Maternal Health in Ethiopia | Text-based confirmation and transmission services via an automated message manager for health extension workers (HEW) | Ethiopia | USAID | 2011 |
| 24 | Ethiopia Health Extension Program | Simple feature phones are used by Health Extension Workers (HEWs) to communicate data into a central operations center using voice. Smartphones over GPRS are also used | Ethiopia | Hopkins | 2012 |
| 25 | Ethiopia mHealth Project | A scalable and comprehensive mHealth platform, with an initial focus on the work flows of HEWs in rural communities by Health Extension Program (HEP) of the Federal Ministry of Health (FMOH) | Ethiopia | Hopkins | 2012 |
| 26 | eVoucher | Marie Stopes International Ethiopia developed an eVoucher system that has enabled the distribution and tracking of vouchers for family planning services to young people using mobile phones | Ethiopia | Hopkins | 2012 |
| 27 | Monitoring Malaria Trends with Cell Phones and Site Visits in Ethiopia | MEASURE Evaluation collaborated for Short Message Service (SMS) technology to build on existing reporting systems to monitor malaria diagnostic, treatment, and control activities | Ethiopia | Hopkins | 2009 |
| 28 | Supply Chain Management System (SCMS) | Data collection and reporting for HIV | Ethiopia | Hopkins | 2009 |
| 29 | Telemed Medical Services | Provides access to reliable, affordable and immediate health advisory and care services through their mobile phones | Ethiopia | CHMI | 2013 |
| 30 | The Last 10 Kilometers (L10K): What it Takes to Improve Health Outcomes in Rural Ethiopia | Mobile phone technology called MagPi (formerly EpiSurveyor), developed by DataDyne for data collection during supportive supervision visits | Ethiopia | USAID | 2008 |
| 31 | The Safe Delivery App | Utilizing smartphones to improve the quality of emergency obstetric and newborn care | Ethiopia | USAID | 2014 |
| 32 | ChildCount+ | SMS text messages to facilitate and coordinate the activities of community health care workers (CHWs) | Ethiopia, Ghana, Kenya, Malawi, Mali, Nigeria, Rwanda, Senegal, Uganda, Tanzania | WHO | 2009 |
| 33 | IQSMS (HIV) | SMS, patient mapping and integrated package of products for HIV/AIDS monitoring | Ethiopia, Kenya, Nigeria, Rwanda, South Africa, Tanzania, Uganda, Zambia | Hopkins | 2009 |
| 34 | Quality of Care Survey | USAID’s flagship Maternal and Child Health Integrated Program (MCHIP) for obstetricians and nurse-midwives to use mobile phones for capturing observational health worker performance data | Ethiopia, Madagascar, Mozambique, Rwanda, Tanzania, Zimbabwe | Hopkins | 2010 |
| 35 | Innovative Approaches to Birth Registration for Maternal Newborn and Child Health | The project also seeks to improve the availability and quality of information on child mortality using innovations such as SMS technologies to increase the number of births being registered | Ethiopia, Mali, Senegal, South Sudan | CIDA | 2014 |
| 36 | OppiaMobile | Mobile delivery of health extension worker training materials | Ethiopia, Nigeria | USAID | 2013 |
| 37 | FHI 360 | Collected data in support of service planning for PEPFAR and other HIV/AIDS care initiatives | Ethiopia, Nigeria, Swaziland | Hopkins | 2011 |
| 38 | eGabon | Advancing the promotion and growth of a local digital ecosystem in Gabon through opportunities arising from the development and roll-out of eHealth applications and services | Gabon | World Bank | 2016 |
| 39 | Zero Mothers Die | Access for pregnant women to appropriate healthcare information, via mobile voice and text messages in local languages and dialects | Gabon, Ghana, Mali, Nigeria, Zambia | WHO | 2014 |
| 40 | BBC Ebola WhatsApp | Ebola public health information service on WhatsApp | Gabon, Guinea, Liberia, Sierra Leone | WHO | 2014 |
| 41 | FrontlineSMS ebola | Hope and life-saving Ebola information text messages sent out by the United Methodist Church twice daily | Gabon, Guinea, Liberia, Sierra Leone | WHO | 2014 |
| 42 | Community Health Nurse on the Go (CNH) | A mobile phone app, provided to community health workers, that combines virtual peer-to-peer support with improved connectedness to a professional network and supervisors | Ghana | CHMI | 2014 |
| 43 | Data Collection for Health Monitoring and for Lobby and Advocacy | District Citizen Monitoring teams, which use mobile data collection tools to reach and make the voices of poor populations heard in remote and difficult locations, and to learn the real impact of the National Health Insurance Scheme (NHIS) | Ghana | WHO | 2014 |
| 44 | Developing a Discrimination Monitoring System | A web-based platform and SMS module to help civil society organization better link with Commission on Human Rights and Administrative Justice (CHRAJ) by promoting advocacy for people living with HIV and key populations | Ghana | Hopkins | 2012 |
| 45 | Early Warning System | USAID-funded Focal Health Regions Project (FHRP) for Early Warning System (EWS), which allows health workers to routinely submit messages to alert decision makers of potential stock-outs of essential medicines in the public health supply chain | Ghana | Hopkins | 2010 |
| 46 | Emergency Contraception Information (EC Info) | Randomized control trial testing the influence of timed delivery of theory-based role model stories on EC users’ family planning intentions via SMS | Ghana | Hopkins | 2010 |
| 47 | Empowering and Mobilizing People Living with HIV/AIDS (EMPower II) | Sending HIV prevention messages via bulk text to voluntary subscribers | Ghana | USAID | 2012 |
| 48 | JSI Early Warning System | Involves the use of mobile phones by health facility staff to report stock levels of 27 tracer commodities (HIV, malaria, and family planning) via SMS | Ghana | USAID | 2011 |
| 49 | LifeLine | Daily ART reminder messages | Ghana | Hopkins | 2012 |
| 50 | mCoaching | Post-training reinforcement, follow-up and mentoring/supportive supervision for maternal and newborn health | Ghana | Hopkins | 2012 |
| 51 | Mobile Information for Maternal Health | Every voice subscriber can opt-in to receive the information on maternal health via SMS and Interactive voice response (IVR) survey questions are embedded into the message | Ghana | USAID | 2013 |
| 52 | Mobile Technology for Community Health in Ghana | “Mobile Midwife” application and Nurses’ Application through MOTECH system | Ghana | Hopkins | 2010 |
| 53 | NO-YAWA | Mobile services to improve reproductive health among youth | Ghana | WHO | 2013 |
| 54 | Participatory Monitoring and Evaluation (PartMe) | A participatory mobile data collection to monitor pro-poor health policies | Ghana | USAID | 2010 |
| 55 | SMS BCC for People Living with HIV/AIDS | As part of the EMPower project, SMS messages are sent to subscribers (usually people living with HIV/AIDS) for information about available treatment and related services, health tips, and other behavior change communication | Ghana | Hopkins | 2010 |
| 56 | Support to the Ebola Virus Preparedness in Ghana | Strengthen government preparedness for early detection and containment of Ebola Virus Disease by diverse activities including installation of hotlines (SMS hotline operators interactive voice response) for the general public and key groups | Ghana | CIDA | 2015 |
| 57 | Text Me! Flash Me! Call me! | HelpLine interventions enable clients to text or ‘flash’ (call and immediately hang-up which is free of charge) or call a HelpLine Counselor (HLC) on duty for HIV/AIDS and other STI information | Ghana | USAID | 2008 |
| 58 | The Ghana Mobile Technology for Community Health (MOTECH Ghana) | 1) Mobile Midwife Application enables pregnant women, new mothers and their families to receive SMS and/or voice messages that provide time-specific information about their pregnancies and childcare each week 2) Community nurses use the Nurses Application to collect patient data and upload records to a centralized database | Ghana | WHO | 2010 |
| 59 | EbolaTxt | Using SMS for social mobilization and reporting on the Ebola outbreak | Ghana, Malawi, Mali, Sierra Leone, Uganda | USAID | 2014 |
| 60 | East Africa MNCH; Southern Africa MNCH; | Envisioned to create a simple, affordable, transferable mobile solution for patient management, data collection and interface to MOH and community clinics | Ghana, Kenya, Malawi, Mozambique, Rwanda, Sierra Leone, Tanzania, Uganda, Zambia, Zimbabwe | Hopkins | 2010 |
| 61 | Mobile Phone Survey Software for End-Use | U.S. President’s Malaria Initiative (PMI) mobile-based End-Use Verification Activity is a quarterly survey to capture information about the malaria supply chain, diagnosis and treatment at public health facilities | Ghana, Malawi, Mozambique, Nigeria, Tanzania, Zambia, Zimbabwe | USAID | 2009 |
| 62 | Global Authentification Network | Selected pharmaceuticals contain a code that's printed on the packaging. By using their mobile phones, individuals can send that code via text or SMS message to the mPedigree system, which quickly returns a message telling them whether or not the medication is authentic | Ghana, Nigeria | CHMI | 2010 |
| 63 | mPedigree | A cloud-based system that tracks pharmaceutical supply chain data on the back end, while allowing buyers to verify the authenticity of medicines for free by text messaging | Ghana, Nigeria | USAID | 2011 |
| 64 | iAfya Mobile health Application | iAfya app will enable health workers in the field to access relevant and up to date health information while on the go, and help to improve service delivery | Ghana, Nigeria, Uganda, Zambia | CHMI | 2012 |
| 65 | MOTECH Suite | A set of services encompassing five key functional mHealth areas: Behavior Change & Demand Generation, Managing Patient Data, Improving Worker Performance, Last-Mile Supply Chain, and Patient Adherence | Ghana, Sierra Leone, Tanzania, Uganda, Zambia | Hopkins | 2012 |
| 66 | Texting4Health | Invite residents of Uganda's second largest commercial city of Jinja to participate in a short health quiz using text messages | Ghana, Uganda | CHMI | 2009 |
| 67 | Guinea Ebola Contact Tracing Application | Contact tracing, supported by a real-time informatics system | Guinea | WHO | 2014 |
| 68 | Multinational Assistance Ebola Project | Deployment of urgency alert and response systems through the use of mobile technology | Guinea, Liberia, Nigeria, Sierra Leone | AfDB | 2014 |
| 69 | HealthMap | The website and mobile app 'Outbreaks Near Me' deliver real-time intelligence on a broad range of emerging infectious diseases | Guinea, Liberia, Sierra Leone | WHO | 2006 |
| 70 | The WelTel mHealth patient engagement service | A two-way text messaging service that connects patients living with HIV or other chronic health conditions to care | Kenya | WHO | 2014 |
| 71 | A Telemedicine-enabled Rural Health Social Franchise | World Health Partners’ teleconsultations using mobile phones, smartphones, video cameras, etc. | Kenya | WHO | 2014 |
| 72 | Access Afya | A social enterprise setting up a network of community care points offering basic healthcare services in low-income neighborhoods -leveraging an SMS follow-up after their visit, appointment reminders and test result notifications | Kenya | CHMI | 2012 |
| 73 | Afya Milele | Health insurance plans that allows subscribers to pay either monthly or annually and can pay their premiums using mobile money transfers, cash transfers or bank transfers | Kenya | CHMI | 2013 |
| 74 | ANC/PMTCT Adherence System | Text messages to coordinate Community Health Worker activities around antenatal care (ANC) | Kenya | WHO | 2010 |
| 75 | Arrow web Hospital | Using computers and mobile phones to input client data and to track health trends and to provide patients with customized health tips and scheduling reminders | Kenya | CHMI | 2007 |
| 76 | Baby Monitor | Interactive voice response technology to offer free mobile screenings to pregnant women and new mothers | Kenya | WHO | 2012 |
| 77 | Chakruok Interactive Radio Program | Text messaging and call-ins were incorporated into the radio drama to encourage listener participation and communication about the RH/FP and HIV issues | Kenya | USAID | 2009 |
| 78 | Chama-Rx | A pooled-purchase subscription program to increase access to safe and affordable drugs at private service delivery points (SDP) in underserved parts; SDP will get funds to invest in a tablet(s), internet connectivity and their first batch of drugs | Kenya | CHMI | 2013 |
| 79 | Changamka Maternal Health Smartcard | The maternal health smartcard is a pre-paid card that allows the bearer to obtain ante-natal, delivery, and post-natal services at listed prices in participating maternity facilities | Kenya | USAID | 2008 |
| 80 | CIC M-BIMA | A platform that allows customers to pay their insurance premium or contribute to their savings using their mobile phones | Kenya | CHMI | 2010 |
| 81 | Clinical Decision Support for CHWs to improve MNCH and OVC care | Community Health workers in Nairobi province use CommCare to support home visits related to MNCH and OVC care | Kenya | Hopkins | 2012 |
| 82 | Flare | Aggregates privately owned ambulance companies into a mobile browser dashboard to help them manage their fleet and an Android app located in the vehicles to respond to patient and hospital requests | Kenya | CHMI | 2016 |
| 83 | FunzoKenya | Use of a mobile phone audio conferencing solution to support e-learning for health workforce | Kenya | Hopkins | 2012 |
| 84 | Gold Star Network Kenya (GSN) | Clients are linked to telecommunications support for treatment adherence for HIV, TB and reproductive health care services | Kenya | CHMI | 2006 |
| 85 | Health at Home/Kenya | Nurses and counselors enter homes with a Android Phone that has a Geographic Positioning System (GPS) app to collect and enter data on family's health, record test results, and document the physical location of the household to guarantee education, counselling and data collection follow-up | Kenya | CHMI | 2009 |
| 86 | Health-E-Net | Remote medical second opinions by linking volunteer specialists, many from the African diaspora, to patients in Kenya via computers, tablets and mobile phones | Kenya | WHO | 2015 |
| 87 | HELP: Health Enablement and Learning Platform | Training and empowering community health workers on basic mobile phones | Kenya | USAID | 2013 |
| 88 | Hnad to Hand Real Care | Integrates physical and virtual (internet and mobile telephony) interventions to increase access to and quality of renal care in resource poor settings | Kenya | CHMI | 2014 |
| 89 | InstAid | Provides a platform for organizations to instantly create, manage, and distribute medical and nutritional e-vouchers via mobile phone technology | Kenya | CHMI | 2013 |
| 90 | Jacaranda Health | Mobile phone communication to encourage positive health-seeking behaviors, and quality improvement systems to standardize the model | Kenya | CHMI | 2010 |
| 91 | Jamii Smart \| KimMNCHip – referrals, mSavings and eVouchers | Strengthening referral system by mobile phone and web portal solution for MNCH services and promoting mSavings and eVouchers for pregnant mothers | Kenya | USAID | 2013 |
| 92 | Jhpiego | Post-procedure follow-up via SMS for appointment reminders and healthy behavior messaging | Kenya | Hopkins | 2012 |
| 93 | M-Afya Kiosks | Allows the clients to pay the community health workers (CHW) via M-Pesa | Kenya | CHMI | 2011 |
| 94 | Malezi-Bora | A mobile application, available in local languages, with audio content regarding maternal and newborn health | Kenya | CHMI | 2011 |
| 95 | MamaKiba | Mobile pre-payment service for customer’s financial planning needs in having a baby | Kenya | WHO | 2008 |
| 96 | Marie Stopes International (MSI) | Empowering patients through ICT client feedback loops in Kenya's health sector | Kenya | Hopkins | 2013 |
| 97 | Maternal and Child Health Integrated Program, Kenya | Using cellular phones to increase return rates for immunization services sponsored by USAID Global Health Bureau’s flagship program, Maternal and Child Health Integrated Program (MCHIP) | Kenya | USAID | 2013 |
| 98 | M-CHANJO | Works by sending automated reminders via SMS to parents to keep then informed on any future immunization dates and appointments for their children | Kenya | CHMI | 2011 |
| 99 | MedAfrica | A free, mobile phone application that allows consumers to access medical information and locate reputable doctors and hospitals near you | Kenya | CHMI | 2011 |
| 100 | Medic Mobile | Improving uptake for ANC and Immunization in Coastal Kenya | Kenya | Hopkins | 2011 |
| 101 | mHBB | Using mobile phones to support Helping Babies Breathe (a simple, inexpensive, evidence-based, and skills-focused program) | Kenya | USAID | 2013 |
| 102 | MitiHealth | An Android application that allows private retail pharmacies efficiently manage their businesses | Kenya | CHMI | 2013 |
| 103 | M-Kifafa | Uses mobile phones to help patients manage their epilepsy and inform decisions made by policymakers in the MOH. | Kenya | CHMI | 2012 |
| 104 | m-Money for Women with Fistula | Transfers of money through M-PESA for bus fare to a nearby hospital to receive Obstetric fistula (O.F) surgery | Kenya | CHMI | 2010 |
| 105 | Mobile App for Management of HIV in Pregnancy | Reducing perinatal transmission of HIV by improving information management | Kenya | USAID | 2015 |
| 106 | Mobile SMS-based Disease Outbreak Alert System (mSOS) | An alert system for outbreaks of yellow fever and Rift Valley fever using pre-defined SMS formats | Kenya | WHO | 2013 |
| 107 | MobiSure | A medical insurance scheme where customers can buy daily premium using their mobile phones | Kenya | CHMI | 2012 |
| 108 | MP3Youth | Implementing a smartphone-based biometric system for participant identification and tracking | Kenya | USAID | 2014 |
| 109 | M-Vaccine | Sends reminder SMS (text) messages to parents when it's time for their children to be vaccinated | Kenya | CHMI | 2012 |
| 110 | National Hospital Insurance Fund (NHIF) | NHIF partnered with Safaricom limited to launch a mobile money platform in its efforts to expand health insurance | Kenya | CHMI | 2010 |
| 111 | Penda Health | Text patients to make sure they're taking their drugs at the right times and in the right way | Kenya | CHMI | 2012 |
| 112 | SANA: Open Source Telemedicine | A reliable tool that allows health workers to transmit any medical file, whether text notes or even information rich x-rays, through a cell phone | Kenya | CHMI | 2009 |
| 113 | The Mobile Health Research Lab: Mobile Wallet | Investigating how mobile money can be used to pre-pay for healthcare | Kenya | USAID | 2010 |
| 114 | TIBU | Data is collected electronically with mobile computer tablets and uploaded into the central database | Kenya | CHMI | 2012 |
| 115 | Totohealth | Social enterprise in Kenya revolutionizing maternal and child health industry by leveraging mobile technology platforms to allow parents and caregivers to monitor the development of their child using the electronic set of Totohealth’s disability indicators | Kenya | CHMI | 2014 |
| 116 | Using mobile phones to support neonatal resuscitation training in Africa (mHBB) | Helping Babies BreatheR (HBB) training is a simple, inexpensive, evidence-based and skills-focused program specifically designed to teach neonatal resuscitation and reduce neonatal mortality in resource-limited areas via web or mobile | Kenya | Hopkins | 2013 |
| 117 | Viva Afya (formerly Carego Livewell) | Uses of two main types of technology, an electronic medical record system (EMR system) and an Early Detection and Prevention System (EDPS) accessible by mobile phone | Kenya | CHMI | 2009 |
| 118 | World Health Partners (WHP) | Providers connect their patients using mobile phones and refer to the catchment facility | Kenya | CHMI | 2008 |
| 119 | ZiDi | A cloud-based software service that is currently optimized for use in dispensaries, health centers and out-patient departments to record and access patient data at any time with web-enabled devices, preferably tablets | Kenya | USAID | 2012 |
| 120 | Nexleaf Analytics | Implementing the sensory technology involving SMS, real-time data collection or GPS in various communities that address water and sanitation hygiene (WASH), vaccines, wildlife conservation, and clean cookstoves | Kenya, Mozambique | CHMI | 2009 |
| 121 | Mobile Health Research Lab | Researches how mobile technology can be used to improve access, quality, and funding of healthcare such as mobile money to pre-pay for healthcare | Kenya, Namibia, Nigeria, Tanzania | Hopkins | 2013 |
| 122 | Mobile Interactions bringing Hope (MI Hope) | Volunteers will be using mobile phones in data collection, sharing information or help in the diagnosis of various ailments | Kenya, Nigeria, South Africa, Zambia | CHMI | 2011 |
| 123 | CliniPAK (Clinical Patient Administration Kit) | The team modified an Open Data Kit (ODK) Collect, an open-source tool for mobile data collection and loaded it onto an Android smartphone that the Community Health Workers (CHWs) carry when visiting patients | Kenya, Nigeria, Tanzania | CHMI | 2011 |
| 124 | East Africa Public Health Laboratory Networking Project | The laboratories in this regional network will serve as surveillance sites to monitor hot spots for disease transmission and will make optimal use of internet and mobile communications to improve public health | Kenya, Rwanda, Tanzania, Uganda | CHMI | 2011 |
| 125 | Mobile 4 Reproductive Health (m4RH) | Automated information to opt-in users on nine different long-acting, short-acting and coitally dependent family planning methods | Kenya, Tanzania | USAID | 2010 |
| 126 | Mobile Diagnostic Services (MODISE) | mHealth diagnostic kit which contains a mobile phone and a collection of in-vitro diagnostic medical device | Kenya, Tanzania | CHMI | 2010 |
| 127 | eCompliance | eCompliance is a system that tracks every dose on a seven inch tablet with a SIM card for TB patients (both counsellor and the patient have to give their fingerprints simultaneously) | Kenya, Uganda | WHO | 2010 |
| 128 | AMREF mLearning | Enables nurses and midwives to use their mobile phones to develop in their profession and keep up to date with latest medical knowledge | Kenya, Uganda | WHO | 2012 |
| 129 | Health eVillages | Provides state-of-the-art mobile health technology including medical reference and clinical decision support resources to medical professionals in the most challenging clinical environments around the world | Kenya, Uganda | CHMI | 2011 |
| 130 | Living Goods | Automated SMS reminders to parents of sick children and pregnant mothers | Kenya, Uganda | CHMI | 2007 |
| 131 | LAUNCH project | Mobile phones are used to improve data flow for better nutrition interventions | Liberia | Hopkins | 2012 |
| 132 | mHero | Connecting and empowering health workers through mobile phones | Liberia | USAID | 2014 |
| 133 | Mobile Health Worker Ebola Response and Outreach | Combining two existing technologies—IntraHealth’s iHRIS software and UNICEF’s mobile phone SMS platform—into a powerful communication tool called mHero (Mobile Health Worker Ebola Response and Outreach) mHero will allow the ministry to instantly send critical information to health workers’ mobile phones during the outbreak and in the future | Liberia | WHO | 2014 |
| 134 | Rebuilding Basic Health Services Project (RBHS) | Routine data collection via Episurveyor | Liberia | Hopkins | 2012 |
| 135 | The Liberian Agriculture Upgrading, Nutrition, and Child Health (LAUNCH) Project | Monitoring food distribution and improving food security | Liberia | USAID | 2010 |
| 136 | Maternova Obstetric Kit | A windable mobile phone charger to call for help or referral | Liberia, Mali, Tanzania, Uganda, Zambia | CHMI | 2010 |
| 137 | Sense Ebola Follow Up | A mobile and internet based contact tracing system used to facilitate data collection and perform real time reporting and GPS tracking | Liberia, Nigeria, Sierra Leone | Hopkins | 2014 |
| 138 | BlueStar Madagascar | A new integrated SMS System, which facilitates voucher verification and payment | Madagascar | CHMI | 2010 |
| 139 | Mobile Finance to Reimburse Sexual and Reproductive Vouchers | Each social franchisee receives 7,500 Ariary (US$3.70) for any contraceptive method and counseling or referral they provide to clients in possession of a subsidized voucher | Madagascar | USAID | 2010 |
| 140 | Using Mobile Finance to Reimburse Sexual and Reproductive Health Vouchers in Madagascar | Providing family planning counseling by distributing the vouchers to eligible clients. It uses mobile phone-based short message service (SMS) money transfer systems to reimburse service providers | Madagascar | Hopkins | 2010 |
| 141 | Action Meningitis | Emergency Triage Assessment and Treatment (ETAT) to triage children when they reach the hospital | Malawi | WHO | 2012 |
| 142 | Child Status Index (CSI) Mobile App | Stand-alone application for orphans and vulnerable children (OVC) committee secretaries to enter information on the wellbing of vulnerable children | Malawi | USAID | 2010 |
| 143 | Chipatala cha pa Foni or Health Center by Phone | A combined hotline and mobile message service to increase access to timely and appropriate maternal, neonatal and child health information, advice and care | Malawi | WHO | 2011 |
| 144 | Christian Aid Scotland (Enhanced Community-Based Maternal, Neonatal and Child Care in Karonga District) | Providing mobile telephones to link communities to their nearest health facilities | Malawi | DFID | 2013 |
| 145 | Community IMCI (cIMCI) | Mobile data service for health surveillance assistants (HSAs) in registering sick children, listening to their complaints, performing an examination, delivering diagnosis, and administering treatment | Malawi | USAID | 2010 |
| 146 | cStock Supply Chains for Community Case Management | Using mobile technology to increase community access to life-saving medicines | Malawi | USAID | 2011 |
| 147 | Hotline by Malawi MOH | Hotline for reducing maternal morbidity and mortality, mobile phone tips and reminder service | Malawi | Hopkins | 2013 |
| 148 | mHealth for Integrated Community Case Management | Improving the quality of child health care at the primary level through integrated decision-support tools | Malawi | USAID | 2012 |
| 149 | Organization For Sustainable Socio-Economic Development Initiative (OSSED MALAWI) | Nutritional, maternal and child health, poor governance systems, drug monitoirng via mobile phone | Malawi | Hopkins | 2010 |
| 150 | Save the Children | District supervisors report summarized data to the national level via sms forms | Malawi | Hopkins | 2012 |
| 151 | Tackling malnutrition of women and children in Africa and Asia | Mobile services for Agriculture and Health with a particular focus on nutrition in collaboration with private sector, government and civil society | Malawi | DFID | 2014 |
| 152 | The Malawi K4Health Mobile Learning Pilot | A distance learning program for community-based health workersto provide up-to-date information in a more efficient manner | Malawi | USAID | 2009 |
| 153 | Association Pesinet | Data entry via mobile phone application to reduce child mortality | Mali | Hopkins | 2009 |
| 154 | Djantoli | Leverages simple mobile technologies as well as community agents to enable remote monitoring by the local doctor | Mali | CHMI | 2008 |
| 155 | Mamans Mobiles contre le Malaria au Mali (MAMMA) | Mobile phones and an app to collect and share data between Community Health Worker’s (CHWs) and health centres | Mali | WHO | 2011 |
| 156 | Monitoring Malaria Trends in Mali Using Cell Phones | MEASURE Evaluation developed a system where malaria-related statistics and information can be accessed via entering a code into their cell phones | Mali | Hopkins | 2012 |
| 157 | o Mobile fleet for health | ANTIM (National E-Health Agency of Mali) supports mHealth applications and reaches the most remote and the least accessible zones | Mali | Hopkins | 2010 |
| 158 | Ma Sante | Mobile data collection on malaria and child health indicators | Mali, Senegal | USAID | 2011 |
| 159 | Grand Challenge Exploration Phase 1 Project | Community health workers using mobile phones to improve maternal, newborn and child health outcomes | Mozambique | USAID | 2010 |
| 160 | Lubombo Spatial Development Initiative | Planning and resource allocation through improved collection, transmission, analysis and management of public health data | Mozambique | Hopkins | 2006 |
| 161 | Maternity Centres of Excellence (MCE) | Project activities include establishing mobile phone networks so that smaller peripheral health facilities can communicate easily with each Centre of Excellence | Mozambique | CIDA | 2014 |
| 162 | mCenas! | SMS client education to improve family planning uptake among youth | Mozambique | WHO | 2013 |
| 163 | mHealth System Strengthening | Linking up the work of community health workers in rural areas with the national health information system through mobile phone technology | Mozambique | DFID | 2015 |
| 164 | MHIN (Mozambique Health Information Network) | Electronic epidemiological surveillance system provides a set of tools for gathering, reporting, storing, analyzing, and alerting epidemic prone diseases at district, province and national level | Mozambique | Hopkins | 2006 |
| 165 | Movercado | Social marketing of public health commodities using basic SMS technology | Mozambique | Hopkins | 2012 |
| 166 | Reliefwatch | A cloud based medical supply and disease tracking platform that uses automated voice calls and simple mobile phones | Mozambique | CHMI | 2013 |
| 167 | SMSaude | A randomised control trial demonstrating the effectiveness of SMS messaging on adherence to HIV care and treatment | Mozambique | Hopkins | 2011 |
| 168 | USAID DELIVER Project | Providing basic smartphones and training to quickly translate hard copy drug consumption data from bubble forms into soft copy data | Mozambique | Hopkins | 2012 |
| 169 | Using SMS messages to improve FP uptake among the youth aged 15-24 | SMS narrative stories (one for boys and one for girls) and an interactive opt-in two way SMS project to ask questions related to key family planning issues for youth | Mozambique | Hopkins | 2013 |
| 170 | CLIP (community Level Interventions for Pre-eclampsia) | CLIP Trial is a cluster randomized controlled trial involving mHealth platforms to reduce pre-eclampsia burden via community mobilization and empowerment of community health workers | Mozambique, Nigeria | Hopkins | 2013 |
| 171 | Pre-eclampsia Integrated Estimate of Risk (PIERS) on the Move | Mobile phone application that assists community health workers in rural, low-resource settings to provide local, rapid and accurate risk assessment, referral, and treatment advice for pre-eclampsia, and transmits information to referral centers | Mozambique, South Africa | USAID | 2012 |
| 172 | inSCALE APE CommCare Application | App for integrated community case management (iCCM) of children with diarrhoea, malaria, and pneumonia | Mozambique, Uganda | WHO | 2014 |
| 173 | Assessing capacity development support for OVC programs | Mobile phones were used to collect the data for a retrospective survey of previous and current partners supporting orphans and vulnerable children (OVC) projects for HIV to understand the outcomes of capacity development support | Namibia | Hopkins | 2012 |
| 174 | Namibia HIV (IntraHealth International, Inc.) | Internet-based client application and SMS to be sent to referred facilitiy and patients for follow up | Namibia | Hopkins | 2013 |
| 175 | Alive & Thrive | Integrating breastfeeding education, cell phones, and microcredit to improve breastfeeding practices | Nigeria | USAID | 2011 |
| 176 | DoctorDial | Allows the patient to have access to confidential Doctor consultation from a remote location via her smartphone, tablets or a personal computer | Nigeria | CHMI | 2016 |
| 177 | DrugStoc | A hybrid eHub of drugs and medical consumables via app, text messages and call center | Nigeria | USAID | 2015 |
| 178 | GxAlert | 3G USB modem and innovative processes integrated into GeneXpert devices to aggregate Multi-Drug Resistant (MDR) tuberculosis tests results in real-time | Nigeria | WHO | 2012 |
| 179 | HiDoctor NG | Brings health care to people through their mobile devices via, chat, audio/video calls | Nigeria | CHMI | 2014 |
| 180 | iDEA: Interactive Distance Education Application | Providing health workers with mobile-based video instruction and reference materials | Nigeria | USAID | 2013 |
| 181 | Kano Connect | The Kano Connect platform digitally connects all health facilities in Kano State by providing phones for over 1,900 healthcare workers, allowing everyone to communicate (using SMS and voice calls) for free within the system and access mobile data | Nigeria | Hopkins | 2014 |
| 182 | Learning About Living | Helps inform and engage young people in Nigeria on issues around sexual health, HIV and AIDS, maternal morbidity and gender violence through a mobile and online platform | Nigeria | CHMI | 2007 |
| 183 | Logistics Indicators Assessment Tool (LIAT) | Supply Chain Management Systems Project (SCMS) using mobile phones with the EpiSurveyor software | Nigeria | Hopkins | 2011 |
| 184 | m4Change + mCCT | Facilitating mobile money transfers to improve maternal and newborn health | Nigeria | USAID | 2014 |
| 185 | m4Change: Clinical Decision Support for facility based ANC providers | SMS reminders and ANC application using CommCare for ANC attendance to women and Community Health workers (CHWs) | Nigeria | Hopkins | 2012 |
| 186 | Mobiles for Quality Improvement (m4QI) | The use of mobile phones to provide post-training support to clinical team members and/or social franchisees in family planning skills. After training, participants will receive quizzes and reminders on their mobile phones in order to re-enforce learning through a remote mechanism | Nigeria | Hopkins | 2013 |
| 187 | MOVE (phasing out it’s previous name; LoMIS) | An open-source last-mile information management platform called Sense -a smartphone application stack to support health workers for workflow facilitation and offline-first data collection | Nigeria | Hopkins | 2014 |
| 188 | Nokia Ovi Life Tools | A Nokia phone application that has a range of personalized informational services, one of which covers health issues such as childcare, fitness, and diseases | Nigeria | CHMI | 2009 |
| 189 | Occupational Health and Safety Managers (OHSM) | Birth and death registration through SMS | Nigeria | Hopkins | 2015 |
| 190 | Omomi (meaning 'my child') | A mobile platform consisting of a mobile app and an SMS service that enables mothers and expectant mothers monitor their children's health, as well as provide access to relevant maternal and child health information plus medical expertise | Nigeria | CHMI | 2014 |
| 191 | Omowunmi | A mobile and web based application that delivers health education via voice calls and text messages to expectant and new mothers | Nigeria | Hopkins | 2014 |
| 192 | Safermom | Uses SMS and voice call to provide vital information to mothers, especially to high risk girls who have been subjected to forced marriages | Nigeria | CHMI | 2014 |
| 193 | SMS Printers to Accelerate Return of Test Results for Early Infant Diagnosis of HIV/AIDS (SMART) | To reduce the turnaround time (TAT) for infant HIV test results using SMS printers | Nigeria | WHO | 2011 |
| 194 | Supportive Supervision (SS) for TB in Nigeria | The use of smartphones during supportive supervision visits at TB care facilities to provide comprehensive monitoring of all clinical, commodity, and laboratory functions. | Nigeria | USAID | 2010 |
| 195 | The SureHealth Plan | SureHealth plan was designed initially to enable subscribers to pay premium through their phones on the mobile money platform | Nigeria | CHMI | 2011 |
| 196 | TrakVac: reminder-recall system for routine immunization | Validating the need for deployment of tracking system using automated Reminder-Recall messages for routine immunization | Nigeria | Hopkins | 2010 |
| 197 | U-Report | Short Message Service (SMS) for subscribers to ask questions and get real time answers about various issues, such as Ebola outbreak | Nigeria | WHO | 2014 |
| 198 | Vaccinator Tracking System (VTS) | A mobile phone-based tracking system for field vaccinators that monitors staff’s coordinates and activities | Nigeria | Hopkins | 2012 |
| 199 | Open Health and Demographic System (OpenHDS) | An open source Android tablet application that enables community field workers to collect pertinent health information | Nigeria, Tanzania | USAID | 2012 |
| 200 | Capacity Development and use of Mobile data collection technology-RWANDA | Through Iformbuilder mobile platform technology together with iPad devices, Oxfam Rwanda programme will fully deploy ICT4D technology in data collection and reporting | Rwanda | DFID | 2016 |
| 201 | Integrated Health systems Strengthening (IHSSP) | SMS text-based, data reporting and recording, CHWs communicating with health facilities | Rwanda | Hopkins | 2009 |
| 202 | mNutrition: Project for Mobile Initiative to deliver nutrition messages | Leveraging available resources to contribute towards the use of mobile technology aimed at positive health and nutrition outcomes and impact to health | Rwanda | DFID | 2015 |
| 203 | RapidSMS and mUbuzima | CHWs report data on newborns and mothers into a national database where health officers at district and national levels can access it and make decisions to improve community health | Rwanda | WHO | 2012 |
| 204 | CRS Senegal mHealth Pilot | Using mobile phones to improve diarrhea case management for children under five | Senegal | USAID | 2013 |
| 205 | Helen Keller International –Senegal | SMS monitoring and evaluation strategies in improving the service delivery of a proven nutritional intervention (Vitamin A supplementation) | Senegal | Hopkins | 2013 |
| 206 | Informed Push Model | Scaling logistics management information systems via push models for increased family planning access and use | Senegal | USAID | 2013 |
| 207 | IVR mLearning Platform in Senegal | Delivering refresher trainings to health care providers by a combination of Interactive Voice Response (IVR) and SMS text-messaging | Senegal | USAID | 2013 |
| 208 | SEDA Automated Health Data Exchange System | Applying mHealth to improve data monitoring | Senegal | USAID | 2011 |
| 209 | Ebola IVR mLearning Course for Health Workers | An Ebola interactive voice response (IVR) course | Sierra Leone | WHO | 2014 |
| 210 | iPhones for Malaria Indicator Survey | For the purposes of the household schedule and to facilitate data entry at the time of the interview on malaria, all household names, village, district and GPS location were recorded into the iPhones | Sierra Leone | USAID | 2013 |
| 211 | mHealth Sierra Leone | Improving maternal, newborn and child health (MNCH), through the development and deployment of mobile data capture solutions | Sierra Leone | DFID | 2011 |
| 212 | Danish Refugee Council mHealth project | Enables citizens standing at a health post in a remote and difficult accessible area to send an SMS short code indicating a need for help | Somalia | CHMI | 2014 |
| 213 | Mobile phone PH application in Somalia | A mobile phone based health promotion application | Somalia | DFID | 2011 |
| 214 | Bettercare (Mhealth training for Nurses and Midwives) | To make accessable their e-learning material across multiple platforms (computer, PDA/tablet, phones) to every nurse and health care practitioner in under-resourced settings | South Africa | CHMI | 2007 |
| 215 | FoneAstra | Cell phone technology to accurately monitor and validate milk temperatures during flash-heating to pasteurize breast milk for human milk banks | South Africa | WHO | 2011 |
| 216 | Hello Doctor | Users receive personal advice from a doctor via website, mobile app,radio, and television, and a call center of registered doctors | South Africa | CHMI | 2007 |
| 217 | JustTested (SMS-Based Support and Information for HIV Testing and Counseling Clients) | SMSs were developed in line with the Health Belief Model for HIV testing and counseling | South Africa | USAID | 2012 |
| 218 | MAMA South Africa | Provides support and information by SMS, mobile web-based community portal, interactive quizzes, social networking platform and pre-recorded weekly voicemail messages | South Africa | USAID | 2013 |
| 219 | Marie Stopes South Africa Mobile Infoline | Mobile Infoline allows individuals to access sexual and reproductive health information via their mobile phone | South Africa | Hopkins | 2011 |
| 220 | m-Assist: Assistance for women undergoing early medical abortion | An RCT evaluating the effect of 3 mobile-based interventions: Support messages to reduce women's anxieties during medical abortion, a questionnaire to self assess completion of the abortion and a mobisite with family planning information | South Africa | Hopkins | 2011 |
| 221 | MHIS (Mobile Health Information System) | Improve the ability of health workers by providing reliable and accurate clinical information at the point of care through the deployment of commercially available, Internet-capable smart phones pre-loaded with a library of pertinent resources | South Africa | Hopkins | 2009 |
| 222 | MomConnect | A pregnancy registry, stage-based messaging and a helpdesk for mothers | South Africa | WHO | 2014 |
| 223 | Owethu Clinics | Mobile clinics using computer, PDA/tablet and phones for real-time assistance with clinical decision making, data collection, communication and financial transactions | South Africa | CHMI | 2013 |
| 224 | Project Khuluma | Creating mobile phone support groups for HIV-infected adolescents | South Africa | USAID | 2013 |
| 225 | Sante Health Platform | Bridging the gap for people who cannot access healthcare services through mobile phone applications | South Africa | CHMI | 2013 |
| 226 | Scaling Up Project Kopano | Online training programme for mentors and a myth buster application for their Kopano project, which provides SMS support groups to women diagnosed with HIV during pregnancy | South Africa | DFID | 2011 |
| 227 | SIMpill® Medication Adherences Solution | A medication management system that monitors the patient’s medication intake and will remind the patient in real time | South Africa | USAID | 2006 |
| 228 | Teen SMS Helpline to Stop Suicide | Allows teens in need of mental heath advice to reach South African Depression and Anxiety Group (SADAG) via SMS | South Africa | CHMI | 2006 |
| 229 | txtAlert for Patient Reminders | txtAlert works with hospital and clinic medical record system (MRS) to remind patients of upcoming appointments | South Africa | USAID | 2007 |
| 230 | Vula Eye Health App | An app to improve eye care in rural areas by enabling healthcare worker to use mobile technology to diagnose eye conditions | South Africa | CHMI | 2013 |
| 231 | Population Services International (PSI) | SMS messages are sent to clients’ mobile phones to remind them of follow-up visits for post-operative review | South Africa, Zambia, Zimbabwe | Hopkins | 2014 |
| 232 | Child Profiling Survey | Mobile data collection technologies using Mobenzi platform for use in a child profiling exercise of 10,244 orphans and vulnerable children (OVC) | Swaziland | USAID | 2013 |
| 233 | Jhpiego-PSI- Combination Prevention Program in the Kingdom of Swaziland | Use of mobile phones for client referrals for clients who test HIV positive | Swaziland | Hopkins | 2011 |
| 234 | Rapid and Effective Action Combating HIV/AIDS | The survey of children to determine the most vulnerable using Mobenzi, a mobile phone service | Swaziland | WHO | 2013 |
| 235 | Accredited Drug Dispensing Outlet | Improving regulatory capacity to enhance pharmaceutical product and service quality | Tanzania | USAID | 2012 |
| 236 | Afya Mtandao | AfyaMtandao promotes the use of ICT in the health sector, by providing a knowledge sharing platform for health workers, raising awareness on ICT for Health, and by providing ICT support services for health institutions | Tanzania | CHMI | 2008 |
| 237 | Aga Khan Development Network eHealth Resource Centre | Works closely with its clients to understand and identify their eHealth needs, providing them with customized, scalable and measurable eHealth solutions involving computer, PDA/tablet, phones and Remote Diagnostic Tool | Tanzania | CHMI | 2011 |
| 238 | Building Synergies in Healthcare (Kujenga umoja katika huduma za afya) | A national-level mobile-optimized e-Learning Platform for public health and health care workers in Tanzania, using an open source MOODLE | Tanzania | WHO | 2015 |
| 239 | Coconut Surveillance | A mobile application that builds on the Malaria Early Epidemic Detection System (MEEDS), an innovative mHealth system used by facilities to report new malaria cases via simple cell phones | Tanzania | Hopkins | 2012 |
| 240 | CommCare for Home-Based Care | The phone-based tool simplifies the collection and transfer of data to a general database and offers decision-making support to HBCPs (home-based care providers) | Tanzania | USAID | 2008 |
| 241 | eFamily Planning (e-FP) | The e-FP application consists of an algorithm (the e-FP job aid) and an SMS-based management tool for family planning | Tanzania | USAID | 2011 |
| 242 | Engage TB | Mobile application that provides a tool for self-screening for tuberculosis | Tanzania | USAID | 2014 |
| 243 | ePartogram | Improving the management of labor and deliveries to reduce maternal and perinatal mortality via tablet-based application and electronic data transmission | Tanzania | USAID | 2015 |
| 244 | Healthy Pregnancy, Healthy Baby or Wazazi Nipendeni | A national Healthy Pregnancy and Safe Motherhood multi-media campaign | Tanzania | WHO | 2012 |
| 245 | ILSGateway | USAID \| DELIVER PROJECT to facilitate routine reporting of supply chain data using short message service (SMS) text messages | Tanzania | Hopkins | 2010 |
| 246 | IntHEC | SMS system aimed at improving uptake of sexual and reproductive health services | Tanzania | DFID | 2012 |
| 247 | mHealth for Community-Based Family Planning Services | Using a mobile application to guide community health workers to provide high-quality family planning services | Tanzania | USAID | 2014 |
| 248 | mHealth for Safe Deliveries in Zanzibar | A mobile phone-based clinical algorithm that takes traditional birth attendants (TBAs) step by step through a screening process to identify pregnant women who are high risk or have any danger signs during pregnancy and labor | Tanzania | USAID | 2011 |
| 249 | mHealth for Safe Deliveries: Ezy Pesa mobile banking service | Mobile application for identifying danger signs during labor and delivery, the phone number of vehicle owners who can transport the patient on an emergency basis, access to money on the phone to pay the vehicle owner for transportation, a record of permissions needed from the family or local decision makers to transport mother and infant in case of emergency, and the ability to contact the referral facility | Tanzania | USAID | 2011 |
| 250 | mHealth for Safer Deliveries | An integrated intervention to improve the quality of care during delivery | Tanzania | USAID | 2011 |
| 251 | Mobile Job Aids for Community Health Workers | Adaptation of an evidence-based FP counseling and screening job aid for use on mobile phones by community health workers as part of an existing HIV home-based care program | Tanzania | Hopkins | 2013 |
| 252 | Mobile Phone Microscopy for the diagnosis of Parasitic Worm Infections | Mobile phone microscope to detect the presence or absence of helminth eggs | Tanzania | USAID | 2012 |
| 253 | MobyApp | Strengthening maternal and newborn health via automated short message service (SMS) reminders to clients, reminding them of upcoming appointments, missed appointments, and approaching delivery dates | Tanzania | USAID | 2013 |
| 254 | Mothers and Infants, Safe, Healthy, and Alive (MAISHA) project | Mobile phone-based tool for service providers to use with pregnant clients | Tanzania | USAID | 2011 |
| 255 | mWater | A free and open-access mobile platform for mapping sites such as water sources, sharing test results and performing surveys | Tanzania | USAID | 2012 |
| 256 | mZinduka! (A Mobile Malaria Community) | Gives opportunity to receive, respond to and engage with Tanzania’s top malaria spokespeople via informative texts, automated calls from pop stars, quizzes and more | Tanzania | WHO | 2012 |
| 257 | NightWatch: Mobile | National Malaria Control Program (NMCP) approves the content of the malaria messages and provides overall guidance on national malaria control priorities via several mobile features including informational messages, quizzes and surveys | Tanzania | USAID | 2012 |
| 258 | OVC data collection | Using mobile phone technology to collect baseline data for an Orphans and Vulnerable Children (OVC) project for HIV | Tanzania | Hopkins | 2012 |
| 259 | Pona na Tigo Bima | Health insurance product offers six tiers of life and hospitalization coverage paid via three monthly installments that are deducted from the customer’s airtime balance | Tanzania | USAID | 2012 |
| 260 | PSI-Ongea Zaidi na Salama | An MIS system using mobile phone SMS that aligns incentives for retailers to purchase condoms and to report these purchases | Tanzania | Hopkins | 2009 |
| 261 | Scaling-up Birth Registration Using Innovative Technology | Scaling up of an innovative birth registration system that quickly uploads records of all birth registrations to a centralized system through text messages sent from mobile phones | Tanzania | CIDA | 2015 |
| 262 | Supporting treatment of childhood malnutrition in Zanzibar (eNUT) | An interactive mobile version of the government-approved treatment guidelines for acutely malnourished children | Tanzania | WHO | 2012 |
| 263 | Tanzania HIV Prevention | Targeted mobile messages regarding male circumcision | Tanzania | Hopkins | 2013 |
| 264 | Tanzania National eVoucher Scheme (Hati Punguzo) | A clinic worker issues an e-voucher to a pregnant woman or an infant’s caregiver via SMS | Tanzania | USAID | 2011 |
| 265 | transportMYpatient | Comprehensive Community Based Rehabilitation in Tanzania (CCBRT) uses mobile phone for phone call and mobile money (M-PESA) to facilitate access to treatment for obstetric fistulae | Tanzania | USAID | 2010 |
| 266 | Voluntary Medical Male Circumcision (VMMC) SMS Information Service | Information about VMMC, where services are offered, and follow-up appointment, wound care, and other reminders | Tanzania | Hopkins | 2010 |
| 267 | Wired Mothers | Wired mothers are pregnant women linked to a primary health care unit through use of mobile phones receiving standard SMS reminders for care appointments and who can call the primary provider in case of acute or non acute problems | Tanzania | WHO | 2009 |
| 268 | ACT for Birth, Uganda | An initiative to improve quality of care at birth using an innovative fetal heart rate monitor (FHRM) and an action-oriented mobile phone-based mortality audit system | Uganda | USAID | 2011 |
| 269 | ASSIST Project | Village Health Teams (VHTs) use mobile phones to support HIV patients | Uganda | WHO | 2013 |
| 270 | BlueStar Healthcare Network, Uganda | Mobile technology applications (mHealth, eHealth, M4QI) and marketing | Uganda | CHMI | 2011 |
| 271 | Clinic Communicator | SMS medication reminders to patients | Uganda | CHMI | 2012 |
| 272 | Connect 4 Change | Smartphone-based patient satisfaction surveys | Uganda | CHMI | 2012 |
| 273 | GATHERdata | Disease surveillance and reporting platform | Uganda | Hopkins | 2008 |
| 274 | Health Child | Audiovisual and mobile-supported content that can be used to inform pregnant women and mothers on maternal and child health issues supported by International Institute for Communication and Development (IICD) | Uganda | WHO | 2007 |
| 275 | iHRIS and Mobile Reference Dictionary | Allows the public to verify that a clinic and/or a medical professional is registered and licensed to practice by sending an SMS message with the doctor’s or clinic’s name to a widely publicized shortcode | Uganda | USAID | 2007 |
| 276 | Improving handwashing practices through SMS | Text to Change (TTC) partnered with USAID through STRIDES for Family Health and Unilever Uganda to promote healthy communities through a Global Hand Washing Day celebration | Uganda | Hopkins | 2011 |
| 277 | JHU∙CCP | SMS program for HIV prevention, family planning etc. | Uganda | Hopkins | 2009 |
| 278 | Living Goods | Mobile messages to deliver targeted health messages, mobile platform for data collection | Uganda | Hopkins | 2012 |
| 279 | Mama Rescue | Mothers use mobile-phone based vouchers to get transportation either from home to health center, or from health center to hospital | Uganda | CHMI | 2014 |
| 280 | MarieTXT | A mobile powered management information system where data is fed into system through SMS and voucher service | Uganda | USAID | 2012 |
| 281 | mHealth for ttC(timed and targeted Counselling) | A Radio Distance learning Approach for continuous learning as well as disseminate desired messages to care givers of children of 0-24 months using mobile phones | Uganda | Hopkins | 2013 |
| 282 | MIRA Channel - Women's Lifeline Channel for RMNCH+A | An integrated mobile phone channel to provide health information to rural women and connect them with public health services | Uganda | Hopkins | 2012 |
| 283 | Mobile 4 Water | Baseline and inspection information collection for water and sanitation | Uganda | WHO | 2011 |
| 284 | Mobile Phones for Improved Access to Safe Water (M4W) | Strengthening monitoring and evaluation of water, sanitation and hygiene via mobile phone | Uganda | USAID | 2011 |
| 285 | Mobiles for Quality Improvement (m4QI) – SHOPS Project | Technology-supported approach to performance improvement including processes for identifying performance gaps in adherence to clinical protocols, a platform to manage and automate the delivery and receipt of text message reminders and quizzes to address the gaps, etc. | Uganda | USAID | 2010 |
| 286 | mTrac | Real-time monitoring of disease prevalence, medicine stock-outs and health service delivery complaints | Uganda | WHO | 2012 |
| 287 | mTRAC Stop Malaria Program (SMP) | MOH’s mTRAC system uses SMS technology to track stock levels of essential malaria medicines at health facilities | Uganda | USAID | 2011 |
| 288 | Reduction of Maternal Mortality Through ICT | Combating maternal and child mortality with text messages | Uganda | USAID | 2011 |
| 289 | Safe Mothers, Safe Babies | An educated public, trained in emergency recognition with cell phone based dispatch system | Uganda | CHMI | 2009 |
| 290 | SMS adherence program for HIV treatment | SMS adherence program, set up by Text to Change (TTC), USAID and Kawempe Home Care Initiative to improve adequate knowledge on HIV treatment and take up of services by sending SMS reminders | Uganda | Hopkins | 2011 |
| 291 | SMS and IVR to Improve Family Planning Services | Through the use of SMS and IVR, the project aims to provide general information on family planning | Uganda | USAID | 2011 |
| 292 | SMS quiz for awareness on Malaria & HIV/AIDS | SMS quizzes and messages are used to improve the uptake of health services of Hope Clinic Lukuli and to improve the knowledge of Hope Clinic Lukuli clients and potential clients | Uganda | Hopkins | 2010 |
| 293 | STRIDES- Strides for Family Health | Mobile technology for health education and behavior change, data management | Uganda | Hopkins | 2009 |
| 294 | The Medical Concierge Group | A consortium of innovative ventures in the Health Care industry involving Call Centres, Clinics, Ambulances, Consultancy, Software Applications and a Foundation | Uganda | Hopkins | 2013 |
| 295 | Tobacco Kills: Say No & Save Lives | Uganda’s first nationwide anti-tobacco campaign utilizing mobile technology and social media by Text to Change (TTC) | Uganda | USAID | 2013 |
| 296 | TTC: SMS to improve HIV awareness | SMS-based multiple choice quiz was administered and rree airtime was offered to users to encourage participation in the program | Uganda | CHMI | 2008 |
| 297 | Uganda Medical and Dental Professional Council (UMDPC) Mobile Directory | An SMS interface to the Uganda iHRIS Qualify system for UMDPC | Uganda | Hopkins | 2011 |
| 298 | Wayo-Nero Strategy | By SMS text messaging, cell phones are used to share mental health information | Uganda | CHMI | 2012 |
| 299 | Winsenga eFHR | A mobile electronic fetal heart rate monitor and corresponding app software that is primarily deployed on Windows Phone | Uganda | CHMI | 2011 |
| 300 | Workplace-based SMS Awareness Campaign | SMS messages on various selected health issues that include family planning as well as HIV counseling and testing are sent to company employees, their families and members of the neighboring communities. | Uganda | USAID | 2009 |
| 301 | Big Heart for Pregnant Mothers Project | Reducing maternal and infant deaths among rural women through provision of accurate health information about postnatal, antenatal, child immunization via SMS | Uganda | DFID | 2014 |
| 302 | ColaLife | ColaLife is a non-profit working with Coca-Cola to supply their Kit Yamoyo which contains crucial ‘social products’ – such as oral rehydration salts, high-dose vitamin A, water purification tablets – to save children’s lives. The scheme uses vouchers, to ensure affordability, and mobile phones for tracking and authentication | Zambia | CHMI | 2008 |
| 303 | Community Led Total Sanitation Mobile Surveillance | Community Champions (CCs) submit village-level water and sanitation data via mobile phone | Zambia | USAID | 2013 |
| 304 | Malaria Community Surveillance For Elimination | A means to sensitively detect malaria infections through community health worker networks and report malaria data by mobile phone, known as a reactive case detection (RCD) protocol | Zambia | WHO | 2012 |
| 305 | Malaria Control Program (MACEPA) | Staff at the clinics report weekly on small carefully chosen data sets that includes the number of tests done, number of positive tests, number of people given malaria treatment and stock information | Zambia | USAID | 2011 |
| 306 | mSpray | An Indoor Residual Spray mapping, monitoring and feedback tool for malaria control | Zambia | USAID | 2011 |
| 307 | POC-IT | National clinical guidelines on HIV/AIDS formatted for offline access on smartphones | Zambia | Hopkins | 2008 |
| 308 | Project Mwana (SMS for Early Infant Diagnosis of HIV) | Using RapidSMS, a free, open-source programming framework to build SMS-based applications, two apps were developed: ‘Results160’ and ‘RemindMi’ | Zambia | USAID | 2010 |
| 309 | Zambia Prevention Care and Treatment Partnership (ZPTP) II | To strengthen enrolment and improve patient retention in both pediatric and adult HIV care by calling back clients for results and missed appointments vis SMS | Zambia | Hopkins | 2011 |
| 310 | Antiretroviral (ARV) Logistics Reporting System | In collaboration with the Ministry of Health and Child Welfare, antiretroviral therapy (ART) facilities use high-resolution camera phones to take pictures of completed Consumption & Requisition Forms, and then send the images to the central level over the GPRS network. | Zimbabwe | Hopkins | 2011 |
| 311 | GSID System for Infectious Disease Surveillance | A mobile health system to read, digitize, and transmit point of care (POC) diagnostic results for important infectious diseases | Zimbabwe | WHO | 2013 |
| 312 | Mobile HIV & Malaria Diagnosis and Reporting System | Android-based software to process, analyze and return rapid diagnostic tests (RDT) results | Zimbabwe | USAID | 2013 |
| 313 | Population Services International (PSI) | Automating data collection for HIV services | Zimbabwe | USAID | 2011 |
| **†**Sources:  WHO- WHO eHealth database [<http://www.who.int/ehealth/resources/who-itu-database/en/>];  World Bank- World Bank Projects & Operations [<http://projects.worldbank.org/>];  AfDB- African Development Bank [<https://www.afdb.org/en/projects-and-operations/>];  USAID- U.S. Agency for International Development [<http://www.africanstrategies4health.org/mhealth-database.html>];  DFID- United Kingdom’s Department for International Development [<https://devtracker.dfid.gov.uk/>];  CIDA- Canadian International Development Agency [<http://www.international.gc.ca/development-developpement/aidtransparency-transparenceaide/browser-banque.aspx?lang=eng>];  Hopkins- mHealth Working Group Inventory of Projects (Johns Hopkins University) [<https://www.mhealthworkinggroup.org/projects/mhealth-working-group-inventory-projects>];  CHMI- Center for Health Market Innovation database [<http://healthmarketinnovations.org/>] | | | | | |
